# An Adaptive S-Method to Analyze Micro-Doppler Signals for Human Activity Classification

**DOI:** 10.3390/s17122769

**Published:** 2017-11-29

**Authors:** Fangmin Li, Chao Yang, Yuqing Xia, Xiaolin Ma, Tao Zhang, Zhou Zhou

**Affiliations:** 1 Department of Mathematics and Computer Science, Changsha University, Changsha 410022, China; taozhang@csu.edu.cn (T.Z.); zhouzhou007@csu.edu.cn (Z.Z.); 2 School of Information Engineering, Wuhan University of Technology, Wuhan 430070, China; lifangmin@whut.edu.cn (F.L.); yqxia@thoughtworks.com (Y.X.); maxiaolin0615@whut.edu.cn (X.M.)

**Keywords:** micro-doppler signal, radar, activity classification, time-frequency analysis, support vector machine (SVM)

## Abstract

In this paper, we propose the multiwindow Adaptive S-method (AS-method) distribution approach used in the time-frequency analysis for radar signals. Based on the results of orthogonal Hermite functions that have good time-frequency resolution, we vary the length of window to suppress the oscillating component caused by cross-terms. This method can bring a better compromise in the auto-terms concentration and cross-terms suppressing, which contributes to the multi-component signal separation. Finally, the effective micro signal is extracted by threshold segmentation and envelope extraction. To verify the proposed method, six states of motion are separated by a classifier of a support vector machine (SVM) trained to the extracted features. The trained SVM can detect a human subject with an accuracy of 95.4% for two cases without interference.

## 1. Introduction

With the development and changes of our society, more and more territory emergency occurred, thus human detection and recognition have gradually become the main research techniques for security and surveillance systems. Aiming for the recognition and classification of human activities, non-contact anthropometric technology can combine human biometric recognition with sensor detection. The detection, recognition and classification of human activity status can better adapt to different application environments in the case of long distance or non carrying. Specifically, radar technology has a lot of advantages such as it will not be influenced by light intensity so that radar can detect targets in low visibility weather and it can penetrate block clothes and so on. Therefore, there is great research value and application significance in human activity detection and feature extraction by using radar technology.

Due to the development of various anti detection technologies, higher requirements for human activity recognition technology are put forward. Therefore, it is necessary to recognize and depict the behavior of the monitored object and the characteristic parameters of the motion more precisely in the realization of human detection. In recent years, the human activity analysis has been focused on the features of micro-Doppler motion. Characteristics of different micro forms such as stride length, stride frequency, and swing angle can be used as a valid basis for moving target classification and identification [1], and exist with a unique form. As a carrier of micro-Doppler motion features, the micro-Doppler signals have individual characteristics in the time-frequency analysis for radar signals, such micro-Doppler motion features can be effectively used to recognize and estimate human activity. Thus, this paper will use continuous wave radar, with micro-Doppler signal as the research object, in three aspects, namely, time-frequency analysis, feature extraction and classification to complete the classification and recognition of human activities.

The traditional Fourier transform is not adapted to the analysis of non-stationary signals, but, in the time-frequency domain, statistical pattern classification of non-stationary signal features is possible. Many techniques can be used for estimation of components of a signal, such as short-term Rényi entropy of its time-frequency distribution [2], wavelets, wavelet packets and the matching pursuit method [3], and adaptive kernel design current method [4]. Recently, based on the imaging algorithm, the S-method approach is presented for the extraction of micro-Doppler features. The proposed S-method can reduce the influence of cross-terms for the echo of each range cell, the effectiveness is confirmed, and the method has low computational complexity in practical use [5,6].

After the concept of micro-motion proposed by Chen in [7], human detection and recognition based on radar technology have great improvement. In [8,9,10], the authors used a continuous wave radar to target human activities, which consist of walking, running, walking while holding a stick, crawling, boxing while moving forward, boxing while standing in place, and sitting still. They introduced deep learning algorithms, directly to a raw micro-Doppler spectrogram for both human detection and activity classification problem. The accuracy results were 97.6% for human detection and 90.9% for human activity classification. Professor Narayanan and his team in [11,12,13] investigated the Hilbert–Huang transform (HHT) method, which was adaptive to nonlinear and non-stationary signals. This study presented simulations of simple human activity, which were subsequently validated using experimental data obtained from both an S-band radar and a W-band millimetre wave (mm-wave) radar. The classification accuracies were obtained at distances of up to 90 m between the human and the radar. However, the existence of endpoint effect limits the practical application of HHT algorithm. It still lacks the value research of the characteristics of human body signals after the completion of the HHT analysis. Orovic et al. in [14,15] proposed a human gait classification technique utilizing the arm positive and negative Doppler frequencies and their relative time of occurrence, by using Hermite multi-window processing technique. Meanwhile, the distribution concentration was shown by using Hermite functions of a few first orders, and it preserved favourable properties of the standard S-method. In order to obtain more echo signals of human body, some researchers embark on equipment improvement. A method of using high frequency bands or increasing radar numbers is proposed. In [16], the dimension of the UWB (Ultra-Wide Band) echoes was reduced using Principal Component Analysis (PCA). In [17], recognition performance between people undertaking the same activity was assessed on a set of experimental data collected via a continuous wave radar operating at X-band using a Naïve Bayesian classifier and a shape-similarity-spectrum classifier. In [18], a feature based on the whole matrix *U* derived from the SVD (Singular Value Decomposition) of the spectrograms has been proposed. It has been shown that high classification accuracy above 98% can be achieved when multistatic data are combined using separated classifiers at each radar node. In [19], the authors focused on the distinction between unarmed and potentially armed personnel, and it has been shown that some feature combinations can provide reduced classification errors when combining data from multistatic aspect angles. On the other side, other combinations were robust in providing low errors even with only monostatic data.

The existing relevant research focusing on the classification algorithm or feature extraction algorithm are relatively simple in time-frequency analysis of micro-Doppler motion signals. Motivated by the above discussions, with the consideration of the potential significance of the Hermite function, in this paper, we study the micro-Doppler motion signals by using time-frequency analysis. The major contributions of our work are as follows:

- For the multi-signal cross interference phenomenon of multi-window processing, the adaptive S-method distribution algorithm is proposed based on a standard S-method, which can guarantee the suppression of multi-signal cross interference based on time-frequency aggregation, thus achieving the purpose of multi signal separation.
- Adopting processing windows of different lengths according to the different signal segments can improve signal aggregation degree, eliminate adjacent signals cross terms and reduce peak oscillation phenomenon at the same time and be helpful to obtain more pure effective motion signals.
- The pendulum arm swing peak matching algorithm of the swing arm is proposed in the process of feature extraction. The algorithm can effectively filter the interference signal by extracting and matching the effective peak value of the swing arm envelope. On this basis, it is more effective to extract relevant feature values.
- The usage of software radio technology to build the system prototype realizes the classification of human movement state. By comparing the experimental results in different environments, the higher accuracy of the system is verified, and the stronger anti-interference performance of the system is verified by interference experiments.

## 2. Experimental Setup

The point scattering model is the simplest model of target electromagnetic scattering excitation. The target can be defined as a three-dimensional reflection function with point scattering characterization, while the occlusion effect can also be achieved in the point scattering pattern. Then, we will analyze human activity process based on point scattering form and extract micro-Doppler motion performance. Mainly starting from non-uniform motion of the human body and swing arm, the scattering point model is used for theoretical derivation of micro-Doppler characteristics induced by the motion, which provides a theoretical basis for subsequent signal analysis.

**Figure 1:**
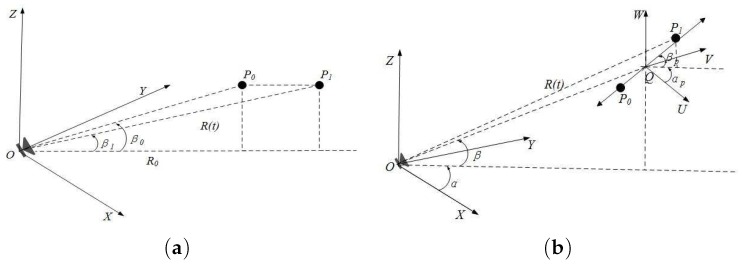
(**a**) spatial phase plane behavior of non-uniform motion object; (**b**) spatial phase plane behavior of the arm swing motion.

In the process of motion, the head and trunk of human body can be considered as a whole because one can keep constant posture in the movement process. In other words, the motion of this part can be simplified as human body center mass motion. The center of mass will have a slight fluctuation in the process of moving, so the motion of the center of mass is modeled as radial horizontal motion. The process is shown in Figure 1a.

As we can see, the radial motion of human body can be characterized by uniformly accelerated motion. If in some period the radial acceleration relative to the radar is designated as *a*, and the body displacement is R(t), its echo signal can be expressed as:

$$\begin{array}{c} S\left(t\right)=A\exp(2\pi j(t-\frac{2R(t)}{C}))=A\exp\left(2\pi jf_{0}t-2\pi jf_{0}\frac{2R(t)}{C}\right). \end{array}$$

$f_{0}$ is continuous wave radar carrier frequency, *C* is the light wave velocity, and the exponential term is phase change induced by the micro-Doppler effect. The micro-Doppler characteristics of the echo can be obtained by separating extraction and derivative operation:

$$\begin{array}{c} f_{\mathrm{md}\_\mathrm{body}}=\frac{1}{2\pi }\frac{d\varphi (t)}{dt}=\frac{2f_{0}}{\cos\beta C}a_{i}t, \end{array}$$

where β is elevation angle between radar and target centroid. Thus, we can see that the micro-Doppler frequency exhibits linearly with elevation fixation, even though the micro-Doppler characteristics are caused by non-uniform motion within a certain time.

When the body is walking, the upper limbs mainly move back and forth periodically around the articulatio humeri and the articulation cubiti, the lower limbs mainly move back and forth around the articulatio coxae and the articulation genus, respectively. It is known from the literature that the motion rules of the human articulatio humeri can be expressed by sinusoidal motion, and the freedom degree of the elbow swing of the human body is related to the speed of walking, which can be defined as a fixed value in the same motion mode. Combined with the experimental scene, the arm swing process is considered as the radial sinusoidal vibration of scattering point, which is shown in Figure 1b.

Then, the micro-Doppler characteristics of the arm swing echo can be expressed as follows:

$$\begin{array}{c} f_{\mathrm{md}\_\mathrm{arm}}=\frac{4\pi f_{a}f_{0}D_{v}}{C}\cos\left(2\pi f_{a}t\right), \end{array}$$

where $f_{a}$ is vibration frequency, and $D_{v}$ is swing amplitude. Thus, the micro-Doppler frequency caused by the target vibration is time-varying, and the change rule is expressed as the forms of sine (or cosine). Moreover, the maximum is determined by the target vibration frequency, amplitude, and radar carrier frequency.

## 3. Multiwindow Adaptive S-Method

Due to the existence of multi-component micro-Doppler motion signals, the noise interference in different environments and the energy intensity and frequency distribution of human activity partial micro-Doppler motion signals are close to each other. The polymerization degree of the signal needs to be further improved for separating the multi-component signals and reducing the adjacent noise interference. The idea of multiwindow proposed by Thomson makes spectral estimation have a good performance in precision and oscillation suppression. Then, Bayram et al. on this basis extended the method of multiwindow spectral analysis to the time-frequency plane [20].

For non-stationary signals, the single window function in spectral analysis is the fundamental cause for not achieving high accuracy simultaneously in both time and frequency domains. Because it is difficult to choose the window function according to the prior rule to satisfy the requirement of time-frequency accuracy, the researchers propose an optimal elliptic symmetric function that has high frequency aggregation in a time-frequency domain. The Hermite function is an orthogonality and ideal aggregation in the time-frequency domain, and it is gathered in a circular region that can be expressed as follows [21]:

$$\begin{array}{c} \left\{\left(t,f\right):t^{2}+f^{2}\leq R^{2}\right\}, \end{array}$$

where *R* is the circle radius. This region can effectively combine the time-frequency domain by placement function. The following is the *k*-th order Hermite function [21]:

$$\begin{array}{c} H_{k}\left(t\right)=\frac{1}{\sqrt{2^{k}k!\sqrt{\pi }}}e^{-\frac{t^{2}}{2}}p_{k}\left(t\right),\;\;k=0,1,2,\cdots , \end{array}$$

where $p_{k}\left(t\right)$ is Hermite polynomial:

$$\begin{array}{c} p_{k}\left(t\right)={\left(-1\right)}^{k}e^{t^{2}}(\frac{d}{dt}{)}^{k}e^{-t^{2}},\;\;k=0,1,2,\cdots . \end{array}$$

Obviously, the above Hermite functions satisfy orthogonality. On account of this, the functions are widely used in signal filtering, speech signal processing and other applications. We adopt the *k*-th order Hermite function into the short time Fourier transform, as a multiwindow function, and the spectral analysis results can be expressed as:

$$\begin{array}{c} MWSTFT\left(t,\omega \right)=\sum_{k=0}^{K-1}d_{k}MWSTFT_{k}\left(t,\omega \right)=\sum_{k=0}^{K-1}d_{k}|\int_{-\infty }^{+\infty }f\left(\tau \right)H_{k}\left(t-\tau \right)e^{-j\omega \tau }d\tau |. \end{array}$$

**Figure 2:**
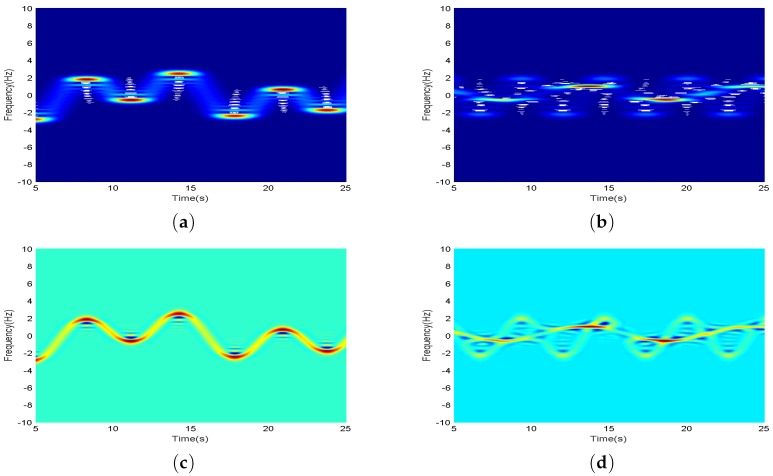
(**a**) is original mono-component signals; (**b**) is original multi-component signals; (**c**) is multiwindow processing results of mono-component signals; (**d**) is multiwindow processing results of multi-component signals.

In order to verify the effectiveness of multiwindow in improving the time-frequency accuracy, simulation experiments are carried out for mono-component signals and multi-component signals, respectively. The signal modes are represented as: $y_{1}\left(t\right)=\exp\left(6\pi j\cos\left(0.1\pi t\right)+4\pi j\cos\left(0.25\pi t\right)\right)$ and $y_{2}\left(t\right)=\exp\left(3\pi j\cos\left(0.15\pi t\right)+jt\right)+\exp\left(4\pi j\cos\left(0.3\pi t\right)-jt\right)$. Figure 2 shows the contrast between the raw signal and the multiwindow signal, where multiwindow adopts up to the 4th order window. We can see that the result is better and the order of complexity is higher when the order is higher.

Compared with the original signal graph, it can be observed clearly from Figure 2c,d that the frequency distribution of the mono-component and the multi-component signals with time-varying improve the aggregation degree in time-frequency domain. Especially, there are many problems when STFT (Short Time Fourier Transform) performs the accuracy of time-frequency analysis in the process of multi-component signals. In Figure 2b, the discrete signal component of the energy distribution is almost covered by another signal component. Two signals have low time-frequency accuracy, and they are seriously aliasing at the intersection of time and frequency. However, the time-frequency signal of mono-components after the multiwindow processing has an oscillation effect at the wave crest, as shown in Figure 2c. The multi-component signal, which is near the effective signal exists cross term effects at the frequency cross of the component signal, and there is obvious oscillation for the signal component with fast frequency conversion, as shown in Figure 2d. On the one hand, the signal components that have poor stationarity are submerged and can not be effectively extracted under the presence of noise interference; on the other hand, the effective signals can be misjudged, which directly affect the separate extraction of multiple components. Therefore, it is necessary to suppress similar cross terms and oscillations in the process of multi signal separation, so as to promote separation of multiple signals.

**Figure 3:**
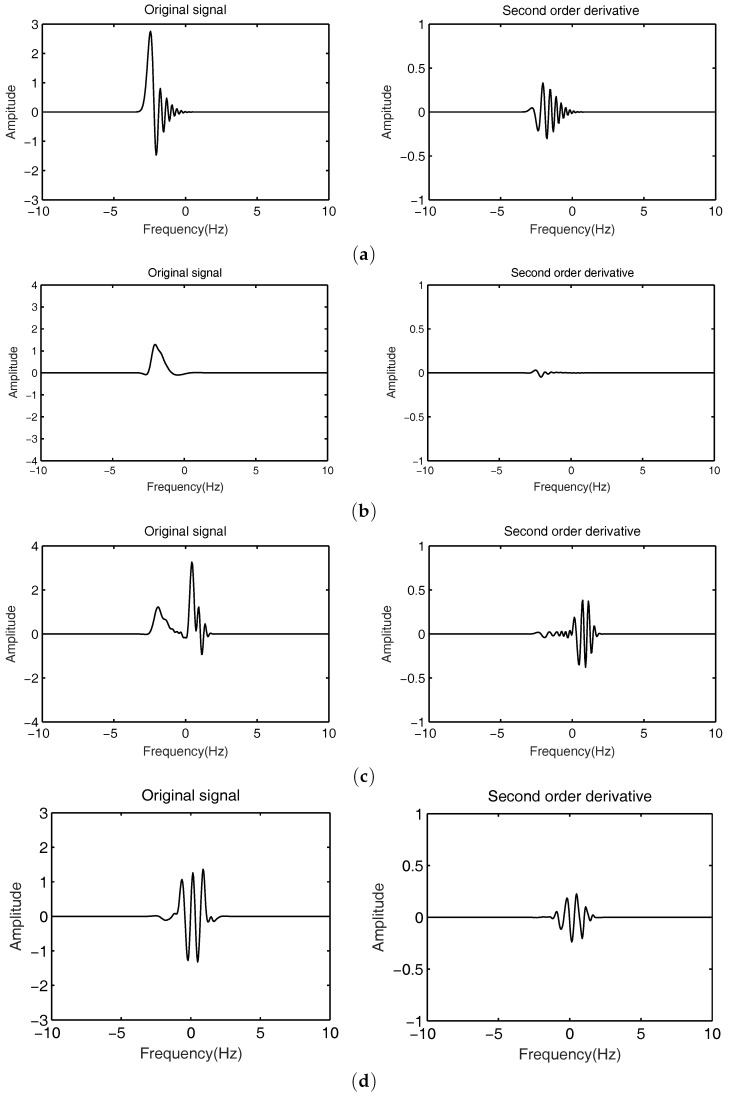
(**a**) is peak amplitude and second order derivative of mono-component signal; (**b**) is falling edge amplitude and second order derivative of mono-component signal; (**c**) is non-cross terms amplitude and second order derivative of multi-component signal; (**d**) is cross terms amplitude and second order derivative of multi-component signal.

For the above two cases, we use second order derivatives for detection and recognition. On this basis, frequency domain windows of different lengths are used to suppress the oscillation and cross term, as shown in Figure 3.

**Figure 4:**
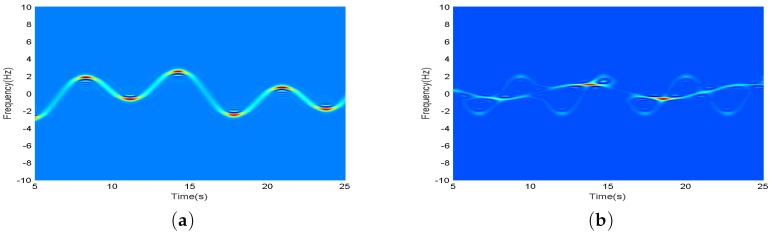
(**a**) is the mono-component adaptive results; (**b**) is the multi-component adaptive results.

Hence, one can see that it can effectively distinguish the oscillation, cross term and gentle signal through the calculation of second order derivative of frequency distribution. We make use of a long window for the signal point approaching 0, which is judged to be a flat signal, and we make use of a short window for the signal point greater than 0, which is determined as an oscillating signal. Therefore, an adaptive S-method (AS-method) is proposed on the basis of multiwindows as follows:

MWAS(n,k)=P·|MWSTFT(n,k)|2+2P·Re∑l=1L(n,k)MWSTFT(n,k+l)·MWSTFT(n,k−l)=P·|∑dkMWSTFTk(n,k)|2+2P·Re∑l=1L(n,k)∑dkMWSTFTk(n,k+l)·∑dkMWSTFTk(n,k−l),

where the second order derivative value are close, so we use fixed window length for gentle signals. For oscillating signals, the window length value is inversely proportional with its second order derivative value and the formula should be changed into a even function. Multiple processing is carried out in order to obtain an effective length value. Suppose that the second order derivative value at the discrete point (n,k) can be calculated as *a*, and the corresponding transformation formula is $L\left(n,k\right)=5e^{-2a^{2}}$. The processing result of the proposed adaptive method i.e., AS-method is shown in Figure 4.

One can see that, for mono-component signal, the oscillation effect near the peak has been corrected and eliminated to a certain extent. For multi-component signals, it can clearly display the changes of the relatively stationary time-frequency signals. Compared with the long window processing results in cross terms, it can preferably suppress the cross aliasing and can clearly observe the frequency change process of the main component. Compared with the short window processing results in non-cross terms, it increases significantly in terms of time-frequency aggregation of signals.

**Figure 5:**
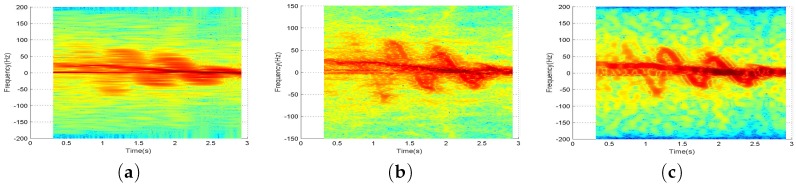
(**a**) is spectrogram; (**b**) is multiwindow processing; (**c**) is AS-method processing.

Therefore, we take the multiwindow adaptive S-method into the time-frequency analysis of radar echo, and take the arm swing of the human body as an example. The signal processing results are shown in Figure 5.

We contrast Figure 5a,b. The time-frequency accuracy of original echo signal is obviously improved after using Hermite multiwindow processing [14,15]. It can clearly distinguish the occurrence time and the variation trend of micro-Doppler signal in the process of arm swing of the human body. On the other hand, it is still unable to separate Doppler signal component and micro-Doppler signal component, due to oscillations occurring in the partial transition regions of two signals because the micro-Doppler signals and noise signals overlap in time-frequency distribution and energy intensity. As for the result of adaptive S-method processing in Figure 5c, one can see that the signals of the trunk and arm swing can be clearly distinguished and the frequency distribution of the two varies with time-varying. For example, near 1.4 s, we can observe the sinusoidal modulation of the independent forward arm swing signal, and the backward arm swing signal near 1.2 s, and we can clearly observe the variation of the component with time-variance. In time-frequency distribution analyses, the multiwindow AS-method can provide more favorable conditions for multi-signal separation.

In order to further verify the improvement in time-frequency aggregation by the multiwindow AS-method, we quantitatively verify the time-frequency aggregation of the three distribution results above by calculating the Renyi entropy and the M value [2], respectively. The larger the Renyi entropy indicates the lower time-frequency concentration of the signal; the higher value of M indicates the higher time-frequency concentration of the signal. The results are shown in Table 1.

According to the signal processing process, the corresponding Renyi entropy is successively reducing, and the value of M is increasing in turn. Thus, the performance of the processed echo signals in time-frequency is improved.

## 4. Effective Signal Extraction

By the multiwindow and AS-method processing, the micro-Doppler signals can separate the effective signal and the noise signal to a certain extent. To extract the pure micro-Doppler signal and further eliminate the interference of noise in signal feature extraction, it is necessary to set a reasonable cutting threshold to filter the noise signal.

**Figure 6:**
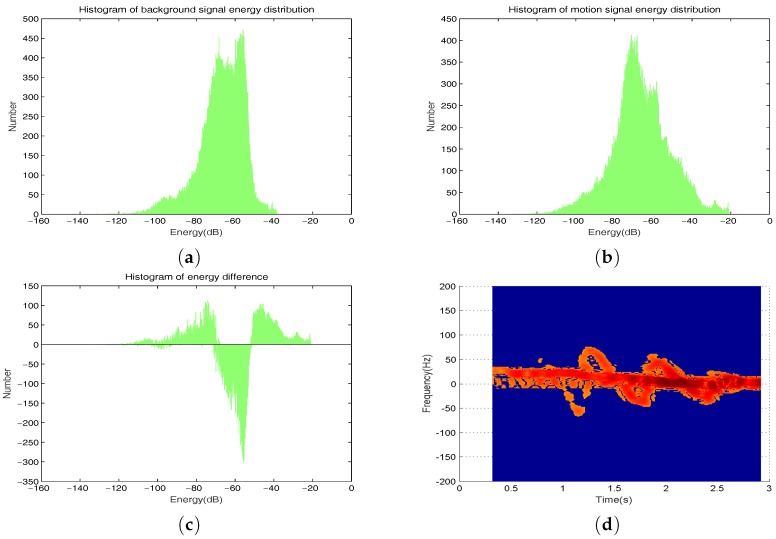
(**a**) is a histogram of background signal energy distribution; (**b**) is a histogram of motion signal energy distribution; (**c**) is a histogram of difference; (**d**) is the result of threshold cutting.

Under different environments, the influence of different motion states on noise distribution can’t be consistent in different environments. Kim and his team in [8] have collected measured data of 12 human subjects performing seven different activities by using a Doppler radar. A support vector machine (SVM) was trained by using the measurement features to classify the activities. In this paper, we use a dynamic threshold cutting method to remove noise interference. On the basis of the existing background noise, the threshold is selected by analyzing the difference of energy intensity between the micro-Doppler signals, as shown in Figure 6.

**Figure 7:**
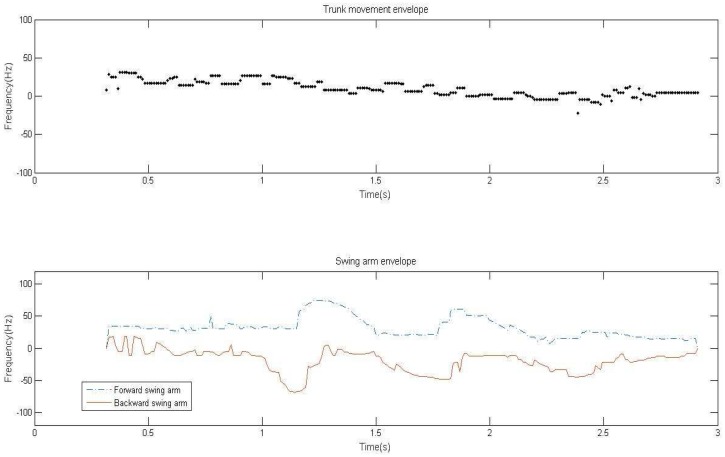
Two types envelop.

In the process of human activity, the trunk is the core part of the echo signal and energy is concentrated in this region. According to the results of the micro-Doppler energy distribution, the envelope caused by trunk motion can be plotted by extracting the frequency distribution corresponding to the maximum energy at each moment. The energy intensity of the micro-Doppler frequency shift signal brought by arm motion is close to interference noise. Energy spectrum still has some scattered noise signal points after the threshold cutting. The square point that is formed at the center of the detection point is an effective proximate signal point, and we can detect the energy of the square point. Moveover, the detection point can be judged as an effective signal point only when the energy values of each effective proximate point of the detection point are greater than the threshold cutting value. On this foundation, the detection of ergodic envelope point is carried out at both ends of the frequency distribution at a certain moment. We take the first effective signal point in the two directions as the envelope point, and then the effective envelope of the arm in different directions is obtained. Trunk movement envelope and arm swing envelope are shown in Figure 7.

Since the human body may walk back and forth, there is positive and negative transformation of Doppler-frequency in the trunk movement envelope, and the arm swing envelope produces relative frequency shift with the trunk movement as the center. In this paper, we use the trunk movement envelope and the arm swing envelope to extract the direction and speed of human activity. The trunk movement envelope can effectively represent the direction and speed of the whole movement of a human body. By selecting other modulation methods or multiple signal sources, it can carry out accurate positioning. The envelope of arm swing can be effectively reflected in the motion of the human body. The characteristic of arm swing in the whole course of motion is similar to a sinusoidal envelope. Through the envelope analysis, we can effectively extract the arm swing period, the arm swing speed and the relative arm swing characteristics, and so on.

## 5. Feature Extraction

In this section, we will classify the states of human activity including sitting still, crawling, running, walking with a single arm, walking with both arms, and walking without arms. In view of the above six different motion states, the following 12 classification features are proposed after fully combining their motion characteristics and differences. Compared to the single use of the motion status parameter in [8,10] and the use of envelope parameter in [15] classification approaches, as shown in Table 2, the classification features proposed in this paper are more elaborated, as shown in Table 3, thus simplifying the classification algorithm. Specifically, by classifying the feature parameters more specifically and explicitly, it helps to describe the states of motion more clearly. As a result, the design of motion classification algorithm is more targeted, and the choice of algorithm flow and threshold is more explicit, and will not lead to ambiguity.

In Table 3, we select three aspects including direct motion characteristics, statistical motion characteristics and relative motion characteristics. In characteristic 1, the trunk Doppler frequency is related to the speed vector of human activity. The different motion states correspond to different motion velocities and directions, and then we calculate Doppler frequency by the mean value method in the whole motion process. In characteristic 2, the Doppler signal bandwidth of the trunk describes the velocity changes of the trunk during the human activity process, which is a result of the micro-Doppler effect of trunk movement. Characteristics 3 and 4 express the maximum deviation of the arm forward swing and arm backward swing. Characteristic 5 shows the swinging speed of arm motion in overall swinging amplitude. Characteristics 6 and 7 illustrate the dispersion degree of arm forward swing and arm backward swing by specific data. Characteristic 8 uses mean square root ratio to describe the relativity of back and forth arm swing. Characteristics 9 and 10 illustrate the dispersion degree of arm swing envelope. Characteristics 11 and 12 are effective arm swing period and effective arm swing time difference. They have a big difference in different states of motion, and it is a key role in the judgment of the arm swing number via combination analysis of this two states.

The above characteristics are completed on the basis of the existing envelope extraction. To obtain a smoother Doppler frequency variation curve, because of the instability of the trunk motion, we will use a least square method to carry out a curve fitting for discrete signal points of trunk. For example, the root mean square error of arm forward swing ($RMSE_{f\_b}$) and the root mean square error of arm forward swing envelope ($RMSE_{\mathrm{front}}$) can be expressed as follows:

$$\begin{array}{c} RMSE_{f\_b}=\sqrt{\frac{1}{N}\sum_{t=1}^{N}(f\left(t\right)-f_{\mathrm{front}}\left(t\right){)}^{2}}\;\;\mathrm{and}\;\;RMSE_{\mathrm{front}}=\sqrt{\frac{1}{N}\sum_{t=1}^{N}{\left(f_{\mathrm{front}}\left(t\right)-\bar{f_{\mathrm{front}}\left(t\right)}\right)}^{2}}, \end{array}$$

where f(t) and $f_{\mathrm{front}}\left(t\right)$ are trunk envelope and the smoothing arm forward swing envelope, respectively. $RMSE_{f\_b}$ can measure the dispersion degree of arm forward swing signal relative to trunk motion. By calculating the mean square error ratio of arm forward swing and arm backward swing, we can quantify the arm swing behavior in human activity. Particularly, it provides methods for the recognition of crawling motion. On the other hand, $RMSE_{\mathrm{front}}$ can measure the dispersion degree of arm swing process and quantify the arm swing amplitude, which is used to distinguish arm swing amplitude in different motion states.

We use the pendulum arm swing peak matching algorithm to calculate the arm swing period and the arm swing time difference. This algorithm consists of two parts: (1) effective peak extraction; (2) peak value matches. The first part is used to obtain the peak value point of the arm swing, the process including peak detection and validity filtering. The validity includes the judgment of deviations between time intervals and amplitudes. The second part is used to match the forward and backward arm swing peak value point and the process includes using a set of sliding pointers to match the effective peak which is judged by peak interval.

## 6. Human Activity Classification

**Figure 8:**
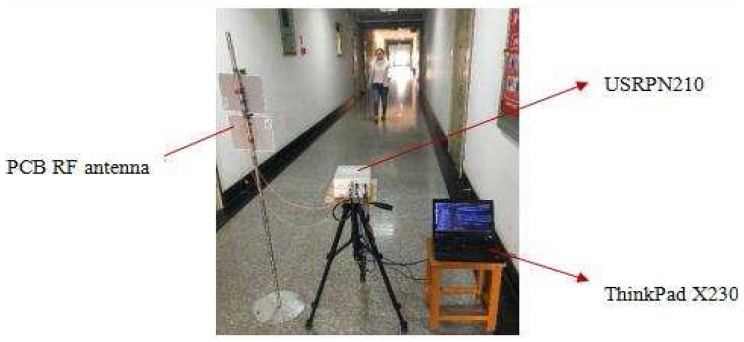
Experimental environment.

In this section, to design a flexible software radio by software radio equipment, we use continuous wave with simple structure and low bandwidth consumption to realize a micro radar monitoring experiment of the human body or other ground targets. The main setting for the experiment is as below: (1) RF (Radio Rrequency) transceiver: we use two PCB (Printed Circuit Board) directional RF antennas, where the transceiver band is 850 MHz–6500 MHz; (2) signal transceiver: we use two USRP (Universal Software Radio Peripheral) N210s [22], one for signal transmission and the other for signal reception, the effective processing frequency band of the built-in transceiver subboard SBX is 440 MHz–4400 MHz, using MIMO (Multiple-Input Multiple-Output) cable connecting two USRP devices to complete the signal transceiver synchronization; (3) signal processing tool: we use a ThinkPad notebook, with a processing speed more than 1000 Mbps, the antenna and USRP are connected by an RF jumper wire, and the computer and USRP are connected by a sixth type cable of PHILPS SWA1964C/93; and (4) the data acquisition software is the open source software radio processing suite GNU Radio 3.7.9. Figure 8 is the experimental environment in corridor.

**Figure 9:**
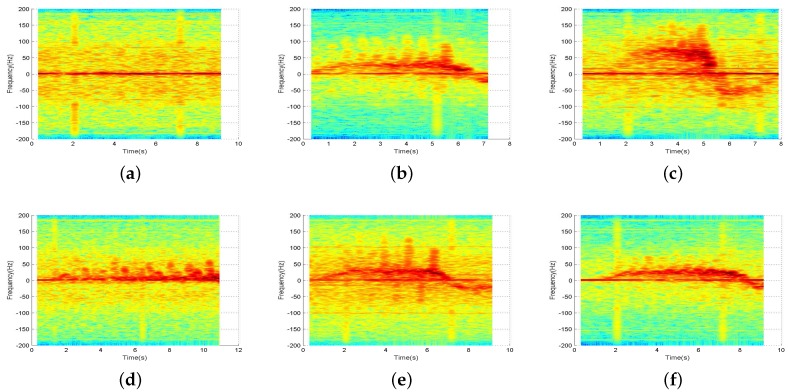
The time-frequency analysis results of six motion states. (**a**) sitting still; (**b**) walking with both arms; (**c**) running; (**d**) crawling; (**e**) walking with a single arm; (**f**) walking without arm.

We take a moving process as a signal collection data sample. Then, we cut the date sample by a fixed starting point and width. A collection process is 15 s, where the first 5 s are static, the middle 5 s are an effective signal segment, and the last 5 s maintain movement to ensure the signal integrity. The time-frequency analysis results of the six motion states are shown in Figure 9.

Due to the influence of the equipment power supply, there is a steady DC signal, which will be eliminated by minimizing the spectrogram. The main signal is divided into two parts. For one part, the signal distribution is concentrated and the intensity is higher, which corresponds to the trunk movement. For the other part, the signals are scattered on two sides of the former part and the intensity is lower. The distribution patterns of different motion states are different, which correspond to the arm movement. In addition to the stationary state, the other five motion states show arm swing micro-Doppler signal distribution near the trunk of the Doppler signal, and different motion states have different micro-Doppler signal frequency distribution and energy intensity, which provides the basis for the classification of motion states by micro-Doppler signals. However, compared with the Doppler signal, the energy distribution of the micro-Doppler signal is discrete and the intensity is lower. The two signals are overlapped in time and frequency domain. The intensity of the micro-Doppler signal, which deviates from the main frequency, is lower, and the intensity and frequency distribution are similar to the signals that are adjacent to noise. In order to extract the micro-Doppler signal effectively, we need to increase the aggregation degree of the signal. The micro-Doppler signal is strengthened by the adjacent signal point, and then the valid signal analysis is extracted.

The envelope analysis of a motion signal is mainly for the analysis of arm swing state. When the signal frequency accuracy is improved, we can extract the envelope of a micro-Doppler signal by an effective threshold value. The envelope analysis consists of two parts: one is envelope statistical feature analysis including the envelope dispersion analysis and relative dispersion analysis. The other one is envelope physics analysis including extraction analysis of effective arm swing period and arm swing interval. The main research work includes three aspects: (1) we analyze relative relation of trunk movement envelope and arm swing motion envelope; (2) we remove interference signal by peak matching algorithm; and (3) we get arm swing characteristic parameters including arm swing period and peak value.

**Figure 10:**
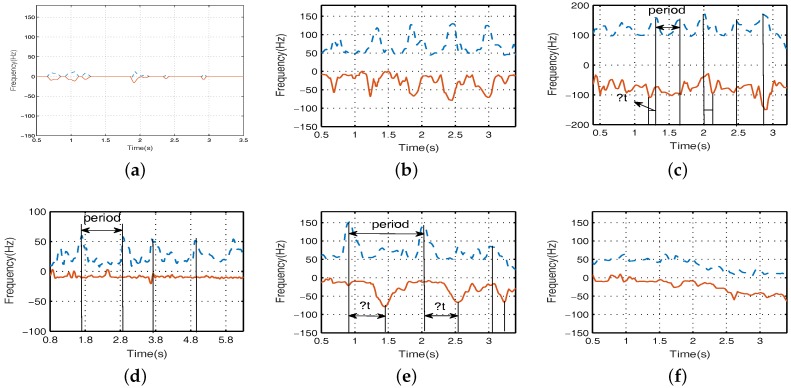
The arm swing envelope analysis of six motion states. (**a**) sitting still; (**b**) walking with both arms; (**c**) running; (**d**) crawling; (**e**) walking with a single arm; (**f**) walking without arms.

In Figure 10, the upper dotted line and the lower solid line are the frequency variation of micro-Doppler signal of arm forward swing and arm backward swing, respectively. ▵t is time difference of arm swing. From Figure 10a,f, we can see that the curves that vary with the Doppler signal are smooth, and no periodic phenomena appears. In Figure 10d, the forward envelope presents periodic peak values, and the backward envelope is fixed frequency value. Therefore, we can distinguish the crawling state effectively by the relativity of the forward and backward envelope. For the cases of Figure 10b,c,e, the forward and backward envelope not only present periodic peak values, but for the corresponding peak pairs, there also exists unstable arm swing time difference. The running frequency distribution is higher, and the walking frequency distribution is lower. Moreover, for walking with a single arm and walking with both arms, the two states can be distinguished by effective arm swing period and arm swing time difference, which needs to further calculate and decompose the envelope by the peak matching algorithm.

Because the way and posture of the human body’s movement is different, in order to guarantee the universality of training model, we collect signals from five individuals (two males and three females), performing six groups of movements and repeating each group 20 times, and there is no human interference during the whole experiment. Remove samples of random clutter due to hardware devices. Each motion state has 100 samples. Then, we gain 600 training samples in total. After signal processing and feature extraction to get six movement states, the mean values of the 12 characteristics are shown in Table 4.

Some characteristic values can illustrate the physical meaning of motion state, such as in characteristic 1. The higher the Doppler frequency, the faster the radial motion of the human body is. Compared to the other five states, the Doppler frequency of running has a high value, and the Doppler frequency value of sitting still and crawling are low. Characteristic 5 is micro-Doppler signal bandwidth, which expresses the speed and amplitude of arm swing in movement. The value of the three states of sitting still, crawling, and walking without arms is smaller than that of the other three states, and it a magnitude difference exists. Characteristic 12 shows arm swing states in the process of movement and the value almost approaches 0 in the two states of sitting still and walking without arms. There are not effective peak pairs that are consistent with different physical features of motion. However, in characteristics 4, 8 and 11, it is hard to distinguish characteristic value, so the six states can not be divided by threshold cutting. Next, we will train and classify the samples by SVM based on decision tree, and verify it by 10-fold cross validation; then, the average recognition accuracy is 95.4%. Table 5 is the corresponding confusion matrix.

We can see from the confusion matrix that the recognition accuracy of crawling is the highest which can reach 100%; the recognition accuracy of sitting still and running is follow, which are 98.7% and 96.2%, respectively; the recognition accuracy of walking with a single arm is the lowest, which is only 85.3%. The reason is that it may be mistaken for walking with both arms. The accuracy of the states of walking process is lower than other states. Moreover, there is cross error in walking with both arms and single arm, with error rates of 5.4% and 13.3%, respectively. The characteristic of walking with a single arm is similar to those of walking with both arms, sitting still and crawling, which leads to lower recognition accuracy.

**Figure 11:**
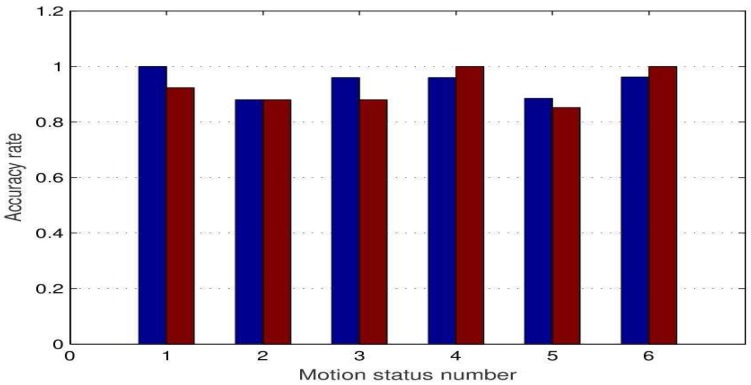
Comparison of indoor(blue) recognition accuracy and corridor(red) recognition accuracy.

Finally, in view of indoor and outdoor environments, the experiments are conducted in enclosed rooms and open corridors, respectively. The contrast results of the recognition accuracy of indoor motion state and corridor motion state are shown in Figure 11.

The X label numbers 1 to 6 correspond to each of the motion states in Table 5. As we can see from Figure 11, indoor and corridor, without interference, the recognition rate of each motion state is very close, and walking with both arms and walking with a single arm have a lower accuracy than other motion states. The classification accuracy of indoor and outdoor motion states was 94.1% and 92.25%, respectively, both of which satisfy basic classification requirements. Moreover, the results show the effectiveness of using a background signal matching method. Under the condition of gathering arm swing environment background signals in advance, this experiment is suitable for indoor and outdoor motion state recognition.

## 7. Conclusions

The application of human activity recognition and classification based on micro-Doppler motion feature has become an area of interest in recent years. In this paper, our study and analysis on the human activity recognition and classification based on micro-Doppler motion have been introduced. The time-frequency distribution characteristics of the micro-Doppler signals have been studied. A multiwindow adaptive S-method distribution was proposed to overcome the shortcomings of existing time-frequency analysis. By using peak matching algorithm, we can extract valid peaks and get peak pairs via the dispersion of the envelope and time interval. The proposed method can eliminate disturbing signals. Then, the effective micro-Doppler signal can be extracted by threshold segmentation and envelope extraction. Combining the characteristics of different activities, we put forward efficient features and apply tree SVM to the human activity classification, which can provide ideal classification accuracy with a simple classified algorithm. Finally, experiments using USRP devices are carried out to validate the performance of classification accuracy and anti-interference.

## Figures and Tables

**Table 1:** The motion signal processing results of Renyi entropy and M value.

Start	Renyi Entropy	M Value
(a) Spectrogram	8.2982	2.9857 × 10${}^{-4}$
(b) Hermite multi-window processing signal	8.1124	3.0335 × 10${}^{-4}$
(c) AS-method multi-window processing signal	7.9581	3.0942 × 10${}^{-4}$

**Table 2:** The motion status parameter.

	Activity	Description
1	Running	The act of running forward quickly by moving arms and legs
2	Walking	The act of walking forward at a moderate speed while moving arms and legs
3	Walking while holding a stick	The act of walking while holding a wooden stick in a horizontal position using both hands
4	Crawling	The act of crawling on both hands and knees while moving forward on the ground
5	Boxing while moving forward	The act of throwing punches using both arms while walking forward
6	Boxing while standing in place	The act of throwing punches using both arms while standing still
7	Sitting still	The act of sitting in a chair with slight fidgeting movement such as shaking of legs, touching of hair, or crossing of arms

**Table 3:** Characteristic parameters.

Number	Characteristic
1	Doppler frequency of trunk movement
2	Doppler signal bandwidth of trunk movement
3	Micro-Doppler frequency offset of arm forward swing
4	Micro-Doppler frequency offset of arm backward swing
5	Micro-Doppler signal bandwidth of arm swing
6	Root Mean Square Error (RMSE) of arm forward swing
7	Root Mean Square Error (RMSE) of arm backward swing
8	Mean square root ratio of arm forward swing and arm backward swing
9	Root Mean Square Error (RMSE) of arm forward swing envelope
10	Root Mean Square Error (RMSE) of arm backward swing envelope
11	Effective arm swing period
12	Effective arm swing time difference

**Table 4:** Characteristic values of six states of motion.

	Sitting Still	Crawling	Both Arms	Single Arm	Without Arm	Running
1	2.8072	3.9998	22.3903	23.9049	23.2223	51.7974
2	48.1358	10.4721	33.5549	34.9253	29.3687	114.1184
3	41.6910	52.4763	74.2836	68.1030	37.4934	82.1812
4	48.0711	29.3298	82.8750	85.3175	42.9847	101.9517
5	39.5960	89.2018	174.8009	165.5351	87.5328	211.2573
6	7.8106	20.7513	31.8811	23.5901	13.5029	34.7365
7	8.5906	10.2654	40.3366	37.9415	22.5313	59.6875
8	0.9369	2.0695	0.7981	0.6382	0.6206	0.6204
9	4.6629	10.7531	19.7651	16.5938	10.3827	26.9559
10	4.6092	3.8733	17.6542	15.0261	11.7459	28.3303
11	0.0177	0.9131	0.5725	0.8770	0.5391	0.4425
12	0.0075	0.0408	0.0858	0.3683	0.0052	0.0699

**Table 5:** Confusion matrix results of interference free classification.

	Sitting Still	Both Arms	Running	Crawling	Single Arm	Without Arm
Sitting still	96.2	0.0	0.0	2.6	0.0	1.3
Both arms	0.0	94.6	0.0	0.0	5.4	0.0
Running	0.0	1.3	98.7	0.0	0.0	0.0
Crawling	0.0	0.0	0.0	100.0	0.0	0.0
Single arm	0.0	13.3	0.0	0.0	85.3	1.3
Without arm	1.4	1.4	0.0	1.4	0.0	95.9
